# Atrazine Induces Reproductive Toxicity in an *In Vitro* Spermatogenesis (IVS) Model

**DOI:** 10.3390/biomedicines13122917

**Published:** 2025-11-28

**Authors:** Monsikan Chaiyakit, Rangsun Parnpai, In K. Cho

**Affiliations:** 1 Embryo Technology and Stem Cell Research Center, School of Biotechnology, Suranaree University of Technology, Nakhon Ratchasima 30000, Thailand; dear_monsikarn@hotmail.com; 2 Department of Environmental Health Sciences, College of Public Health, University of Georgia, Athens, GA 30602, USA; 3 Regenerative Bioscience Center, University of Georgia, Athens, GA 30602, USA

**Keywords:** atrazine, human induced pluripotent stem cells, *in vitro* spermatogenesis, Nrf2 signaling pathway, oxidative stress, reproductive toxicity, Huntington’s disease

## Abstract

**Background/Objectives:** Atrazine (ATZ) is a widely used herbicide, and most studies of its reproductive toxicity have been conducted *in vivo* using animal models, where ATZ disrupts redox homeostasis, leading to male reproductive dysfunction. However, its molecular mechanisms of action in human spermatogenic cells remain poorly understood. Huntington’s disease (HD), an autosomal dominant disorder caused by abnormal CAG repeat expansion in the *HTT* gene, exhibits heightened oxidative stress sensitivity and mitochondrial dysfunction, which may further impair reproductive function. This study investigated ATZ effects on human spermatogenesis using an *in vitro* spermatogenesis (IVS) model derived from human induced pluripotent stem cells (hiPSCs), focusing on Nrf2-mediated oxidative responses and apoptotic regulation during spermatogonial stem cell-like cell (SSCLC) differentiation in wild-type (WT) and HD hiPSC lines. **Methods:** Two WT and two HD hiPSC lines carrying 44 (HD1) and 180 (HD2) CAG repeats were treated with ATZ (0, 0.01, 1, or 10 μM) for 30 days, followed by differentiation into SSCLCs for 15 days under continuous exposure. Expression of pluripotency (*OCT4*, *SOX2*), oxidative stress (*NFE2L2*, *SOD1*, *GPX1*, *NQO1*), cell cycle (*CDK1*), apoptosis (*BCL2*, *BAX*, *CASP3*, *CASP9*, *FAS*, *FASLG*), and spermatogenic markers (*DAZL*, *ZBTB16*, *GFRA1*, *PIWIL2*) were assessed by immunocytochemistry and qRT-PCR. **Results**: Long-term ATZ exposure affected pluripotency markers in hiPSCs and SSCLC differentiation in a cell line–dependent manner. WT cells exhibited early differentiation suppression without significant apoptosis. HD1 cells were highly sensitive: low ATZ doses (0.01–1 μM) partially activated intrinsic and extrinsic apoptotic pathways, whereas high-dose ATZ (10 μM) reduced Nrf2-target and spermatogenic gene expression, strongly impairing SSCLC maturation. HD2 cells showed pronounced oxidative stress with robust Nrf2-driven antioxidant responses and *BCL2* that supported differentiation at low doses. However, excessive oxidative or proliferative signaling, including *CDK1* upregulation at high ATZ concentrations, disrupted redox balance and SSCLC differentiation in HD2 cells. **Conclusions**: ATZ exerts dose- and genotype-dependent effects on IVS through coordinated regulation of oxidative stress and apoptosis. These findings highlight the interplay between Nrf2-mediated antioxidant defenses, apoptotic signaling, and genetic background in shaping spermatogenic outcomes, providing mechanistic insight into ATZ-induced reproductive toxicity in a human-relevant *in vitro* spermatogenesis model.

## 1. Introduction

Environmental contaminants have become a growing concern for human and animal health. Among these, endocrine-disrupting chemicals (EDCs) have attracted particular attention due to their ability to interfere with hormone signaling and impair reproductive function [1]. Atrazine (ATZ), a triazine-based herbicide widely used for agricultural weed control, has been identified as a potential EDC. Although effective for weed control, its environmental persistence and mobility raise concerns, as exposure has been linked to reproductive and developmental abnormalities in humans [2,3,4]. Biomonitoring and environmental monitoring studies indicate that environmentally and physiologically relevant ATZ levels are typically in the tens to hundreds of ng/L range in surface and drinking waters, with the U.S. EPA drinking-water maximum contaminant level (MCL) set at 3 µg/L (14 nM), and in the low ng/mL range (5–50 nM) for ATZ and its metabolites in human urine and seminal plasma [5,6,7,8].

Mechanistically, ATZ toxicity is closely associated with oxidative stress, which occurs when the balance between reactive oxygen species (ROS) production and antioxidant defenses is disrupted [9,10,11]. Excessive ROS can disrupt redox homeostasis, leading to oxidative damage and cellular dysfunction. A key regulator of antioxidant defense is the nuclear factor erythroid 2–related factor 2 (Nrf2) pathway, which governs the expression of genes involved in ROS detoxification and cellular protection. Dysregulation of Nrf2 pathway exacerbates oxidative injury, compromises DNA integrity, and impairs cell survival, thereby sensitizing cells to further stress [12,13,14]. In parallel, ATZ activates the DNA damage response (DDR) and modulates apoptotic signaling, both essential for genomic stability and normal cell function [15]. Disruption of these interconnected pathways can lead to abnormal cell survival or death, ultimately resulting in tissue damage and impaired organ function.

ATZ-induced reproductive toxicity has been reported across diverse species. In males, it interferes with testicular steroidogenesis by impairing Leydig cell function, reducing testosterone synthesis, and inducing apoptosis, resulting in decreased sperm count and compromised semen quality [16,17,18,19]. Maternal exposure studies show that ATZ crosses the placenta and is transmitted through breast milk, causing DNA damage and mitochondrial dysfunction in offspring germ cells [20,21]. Beyond mammals, ATZ exposure impairs reproduction in aquatic, amphibian, and invertebrate species by inducing testicular degeneration and downregulating genes involved in spermatogenesis, antioxidant defense, and DNA repair [22,23,24]. Although some studies report minimal effects at low exposures, such variability likely reflects differences in species, developmental stages, and exposure conditions [3,25]. These conflicting findings underscore the complexity of ATZ’s biological effects and highlight the need for human-relevant experimental systems to clarify its molecular mechanisms.

Most previous studies have examined acute or high-dose ATZ exposures, which may not accurately reflect environmentally relevant concentrations or chronic effects in human systems. This limitation is particularly critical for human reproductive health, as spermatogonial stem cells (SSCs) are highly proliferative and essential for maintaining spermatogenesis and male fertility [26]. Direct evaluation of ATZ effects on human spermatogenesis has been restricted by the absence of suitable models. Recent advances in human induced pluripotent stem cells (hiPSCs) technology now enable the differentiation of hiPSCs into spermatogonium-like cells (SSCLCs), offering a powerful platform to investigate how environmental toxicants and genetic factors influence human germline development [27].

Huntington’s disease (HD) is an autosomal dominant neurodegenerative disorder caused by an expanded and unstable cytosine–adenine–guanine (CAG) trinucleotide repeat in the huntingtin (*HTT*) gene [28]. The degree of CAG instability varies by cell type and developmental stage, and oxidative stress has been implicated in both repeat expansion and disease progression [28,29,30,31]. Recent evidence indicates that oxidative stress may drive somatic CAG repeat instability [32,33,34]. Given ATZ’s known ability to induce oxidative stress, environmental exposure could potentially influence trinucleotide repeat stability and spermatogenic processes warrants further investigation.

To address this gap, we utilized an established human *in vitro* spermatogenesis (IVS) model. Both WT and HD hiPSC lines carrying distinct CAG repeat expansions were differentiated into SSCLCs under ATZ exposure. This approach enabled the evaluation of the impact of sustained, low-dose ATZ on oxidative stress, Nrf2 signaling, and apoptotic control throughout SSCLC differentiation. Overall, this study provides new insights into how chronic ATZ exposure may disrupt molecular pathways critical for germline maintenance and differentiation, potentially contributing to reproductive dysfunction in human-derived systems.

## 2. Materials and Methods

Human induced pluripotent stem cell culture: Two WT hiPSC lines (BJ and ND41658) and two HD hiPSC lines (ND38547 with 44 CAG repeats (Q), HD1; and ND36999 with 180 Q, HD2) were used in this study. HD1 represented adult-onset HD with a moderate CAG expansion, whereas HD2 represented juvenile-onset HD with a very large expansion, allowing us to evaluate CAG length–dependent cellular responses to ATZ exposure. The BJ line was kindly provided by C.A.E., and the other lines were obtained from the NINDS Human Cell and Data Repository. All hiPSC lines were maintained on Matrigel-coated (Corning, NY, USA) dishes in mTeSR™ Plus medium (STEMCELL Technologies, Vancouver, BC, Canada) at 37 °C under 5% CO_2_. Medium was replaced every other day, and cells were passaged every 4–7 days using ReLeSR™ (STEMCELL Technologies, Vancouver, BC, Canada) onto freshly Matrigel-coated dishes.

Atrazine (ATZ) preparation and exposure: ATZ (PESTANAL^®^, Sigma-Aldrich, St. Louise, MO, USA) was dissolved in dimethyl sulfoxide (DMSO) to prepare a stock solution. Working concentrations were freshly diluted by culture medium before each medium change to final concentrations of 0.01, 1, or 10 µM ATZ, resulting in a final DMSO concentration of ≤0.1% in all ATZ-treated groups. For long-term exposure, hiPSCs were continuously cultured with ATZ for 30 days. At the end of this period, cell pellets were collected for subsequent analysis, while a subset was further subjected to *in vitro* SSCLC differentiation.

*In vitro* differentiation of hiPSCs into spermatogonial stem cell-like cells (SSCLCs): IVS was performed following our established protocol [35,36,37], with medium conditions adapted from [38]. After 30 days of ATZ exposure, hiPSCs were passaged using ReLeSR™ onto mitomycin C-treated STO feeder cells (Sandos inbred mouse [SIM]-derived, resistant to 6-thioguanine and ouabain, supplemented with leukemia inhibitory factor [LIF]). After 2 days, when hiPSCs reached 80–90% confluence, mTeSR™ was replaced with SSC differentiation medium consisting of α-MEM with L-glutamine (Invitrogen, Waltham, MA, USA), 200 µg/mL ascorbic acid (Sigma-Aldrich), 3% knockout serum replacement (KSR; Invitrogen), 1 ng/mL human basic fibroblast growth factor (hbFGF; BD Biosciences, San Diego, CA, USA), 20 ng/mL glial-derived neurotrophic factor (GDNF; R&D Systems, Minneapolis, MN, USA), 1% lipid mixture 1 (Sigma-Aldrich), 1× ITS Premix Universal Culture Supplement (Corning), and 0.5× penicillin/streptomycin (Invitrogen). ATZ supplementation was continued throughout differentiation, with fresh medium replaced every 2 days. Cells were differentiated for 15 days, and pellets were collected at days 5 and 15 for characterization. Morphological changes were documented using an Olympus bright-field microscope. All experiments were performed in triplicate (n = 3 biological replicates). Each replicate represents a separate SSC differentiation performed in a distinct culture dish.

Immunocytochemistry (ICC): To evaluate the expression of pluripotency and germ cell-associated proteins, cells were washed three times with phosphate-buffered saline (PBS; Lonza) and fixed with 4% paraformaldehyde (PFA) for 30 min at room temperature. Then cells were permeabilized with 0.2% Triton X-100 solution for 15 min and blocked with 2% bovine serum albumin (BSA; Sigma-Aldrich) and 5% normal goat serum (NGS) for 1 h. Cells were sequentially incubated overnight at 4 °C with primary antibodies against OCT4 (1:500; Santa Cruz Biotechnology, Dallas, TX, USA), SOX2 (1:500; STEMCELL Technologies), PLZF/ZBTB16 (1:200; R&D Systems), and PIWIL2 (1:200; Santa Cruz Biotechnology). After three PBS washes, cells were incubated for 1 h at room temperature with Alexa Fluor^®^ 488 or Alexa Fluor^®^ 594 conjugated secondary antibodies (1:1000; Molecular Probes, Eugene, OR, USA). Nuclear DNA was counterstained with Hoechst 33342 (1:1000; Molecular Probes) for 10 min. Fluorescence images were captured using an Olympus BX51 microscope and processed with CellSens software version 1.17 (Olympus Inc., Center Valley, PA, USA). The mean levels of fluorescence intensity were measured by analyzing the FIJI software version 2.16.0.

Quantitative Real-Time PCR (qRT-PCR): To investigate the effects of ATZ on hiPSCs and during IVS, pellets from all hiPSC lines (after 30 days of ATZ exposure) and from differentiated cells at day 5 and day 15 were collected for analysis. Total RNA was extracted from cell pellets using TRIzol reagent (Invitrogen), followed by DNase treatment with the Turbo DNA-free™ Kit (Invitrogen). For each sample, 500 ng of RNA was reverse transcribed into cDNA using the iScript™ cDNA Synthesis Kit (Bio-Rad, Hercules, CA, USA). qRT-PCR was performed with iQ™ SYBR^®^ Green Supermix (Bio-Rad) on a CFX96™ Real-Time PCR Detection System (Bio-Rad). Relative gene expression was calculated using the ∆∆Ct method. *GAPDH* served as the endogenous control, and expression levels were normalized first to *GAPDH* and then to the untreated control group for each cell line and differentiation stage. All primers were shown in Supplementary Table S1.

Statistical Analysis: Unless otherwise stated, statistical comparisons were performed using one-way ANOVA followed by Tukey’s multiple comparison test. Analyses were conducted using GraphPad Prism InStat version 8 (GraphPad Software, Inc., San Diego, CA, USA). Data are presented as mean ± standard error of the mean (SEM). Statistical significance was defined as *p* < 0.05.

## 3. Results

### 3.1. Directed Differentiation Confirmation of WT and HD-hiPSCs into SSCLCs In Vitro

**Figure 1:**
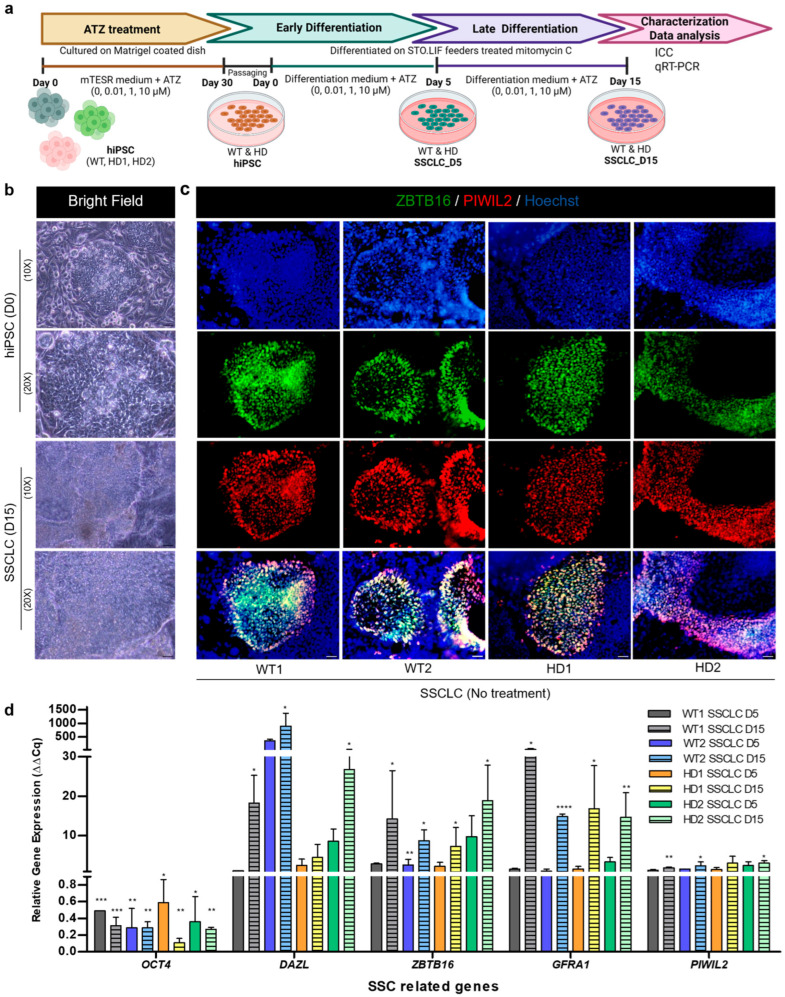
Directed differentiation confirmation of WT and HD-hiPSCs into SSCLCs *in vitro*. (**a**) Schematic representation of the ATZ exposure and SSCLC differentiation model from hiPSCs (with and without ATZ treatment). (**b**) Bright-field images at day 0 and day 15 of differentiation (scale bar = 100 μm). (**c**) ICC images at day 15 showing expression of PIWIL2 and ZBTB16 in all cell lines (scale bar = 100 μm). (**d**) qRT-PCR analysis showing relative gene expression profiles after SSCLC differentiation. Both WT and HD cell lines exhibited a significant decrease in pluripotency markers (*OCT4* and *SOX2*) and an upregulation of SSC markers such as *DAZL*, *ZBTB16*, *GFRA1*, and *PIWIL2* from day 5 to day 15 of differentiation. Data are presented as mean ± SEM from three biological replicates (n = 3). Statistical significance was determined by one-way ANOVA followed by Tukey’s multiple comparison test. Comparisons were made with the hiPSC state of each cell line: * *p* < 0.05, ** *p* < 0.01, *** *p* < 0.001, **** *p* < 0.0001.

Throughout the ATZ treatment and differentiation process (Figure 1a), morphological changes were observed. By day 15, these clusters further developed into smaller cellular forms, suggesting continued morphological progression (Figure 1b). Immunocytochemistry confirmed germ cell-specific marker expression. Differentiated cells showed co-expression of ZBTB16 and PIWIL2 (Figure 1c). Notably, PIWIL2-positive staining was more widespread than ZBTB16, consistent with PIWIL2 being expressed in early spermatocytes [39]. qRT-PCR analysis further validated germ cell induction (Figure 1d). *OCT4* mRNA, highly expressed in undifferentiated hiPSCs, was progressively downregulated after differentiation, reaching its lowest level at day 15 (*p* < 0.01). *DAZL* expression increased steadily from day 5 through day 15 (*p* < 0.05). Both *ZBTB16* and *GFRA1*, key spermatogonial stem cell markers, were significantly upregulated at the 15-day differentiation period. *PIWIL2* expression also showed significant upregulation from the hiPSC stage to day 15. These consistent changes in morphology, protein expression, and gene expression indicate the successful differentiation of both WT and HD-hiPSCs into germline-like cells, exhibiting characteristics of early spermatocytes or SSCs.

### 3.2. Comparison of SSC Related Gene Expression Between WT and HD-hiPSCs at Day 15

**Figure 2:**
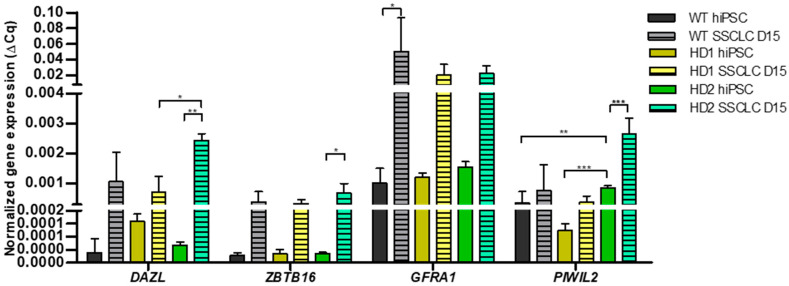
Comparison of relative SSC gene expression profiles between WT and HD hiPSCs during differentiation into SSCLCs. qRT-PCR analysis shows that, under ATZ untreated conditions, SSC gene expression profiles after SSCLC differentiation did not differ significantly between WT and HD lines. Both cell types exhibited upregulation of SSC markers, including *DAZL*, *ZBTB16*, *GFRA1*, and *PIWIL2*, during the transition from hiPSCs to SSCLCs. Comparisons were made with the hiPSC state of each cell line. Statistical significance was determined using one-way ANOVA followed by Tukey’s multiple comparison test: * *p* < 0.05, ** *p* < 0.01, *** *p* < 0.001.

Alterations in spermatogenesis and sperm function have been reported in both animal models and human males with HD [40]. To evaluate whether mutant huntingtin (mHTT) affects SSCLC differentiation, we compared the expression of key SSC markers in WT and HD-hiPSCs at day 15 using qRT-PCR. The expression levels of early SSC markers, *DAZL* and *ZBTB16*, were comparable between WT, HD1, and HD2 lines (Figure 2). Notably, HD2 cells exhibited the highest *PIWIL2* expression (*** *p* < 0.001), a late SSC marker, indicating efficient progression toward SSCLCs compared with their parental hiPSCs. These results indicate that mHTT selectively affects SSCLC differentiation in a cell line–dependent manner, despite most SSC markers remaining largely unaffected.

### 3.3. Atrazine Exposure Alters Pluripotency in Human iPSCs

**Figure 3:**
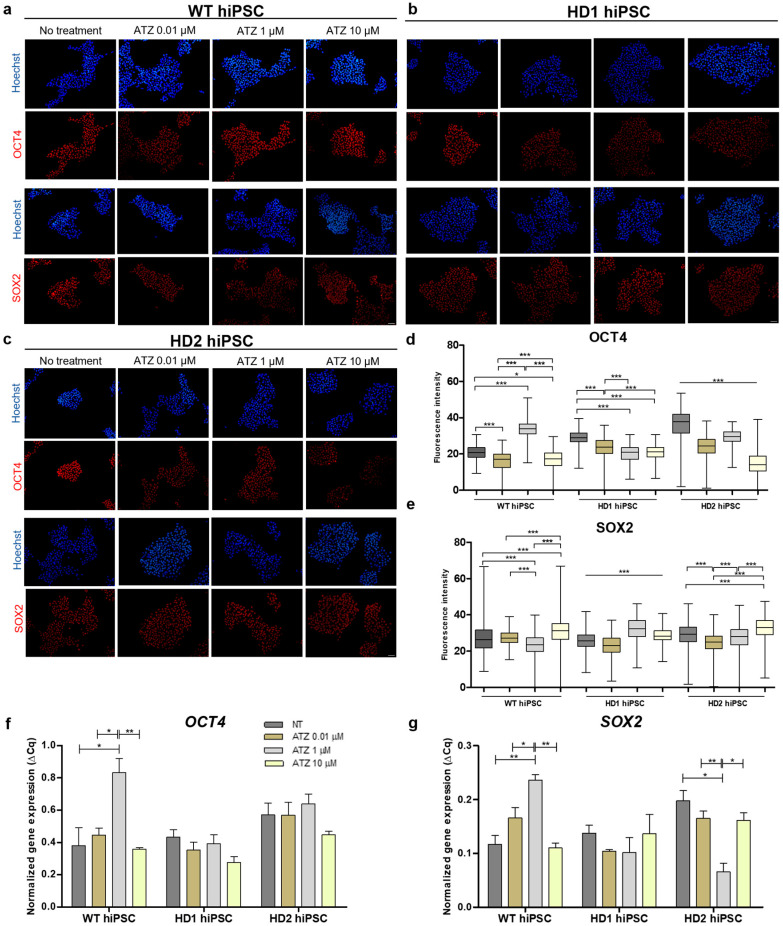
Effect of long-term ATZ exposure on pluripotency in hiPSC state. (**a**–**c**) Immunofluorescence images of WT, HD1, and HD2 hiPSCs after 30-day ATZ exposure, showing concentration- and cell line–dependent alterations in pluripotency markers (OCT4 and SOX2). (**d**) Quantification of ICC showing ATZ-induced downregulation of OCT4 staining intensity, particularly in HD1 and HD2. (**e**) SOX2 staining intensity following ATZ exposure. (**f**,**g**) qRT-PCR analysis showing alterations of *OCT4* and *SOX2* expression after ATZ exposure, particularly in HD1 and HD2, although most of the changes were not statistically significant. Statistical significance was determined using one-way ANOVA followed by Tukey’s multiple comparison test: * *p* < 0.05, ** *p* < 0.01, *** *p* < 0.001.

Since ATZ can induce excessive ROS production, which may impair self-renewal and cause stem cell exhaustion [41], we investigated whether long-term ATZ exposure affects hiPSC pluripotency. ICC analysis of OCT4 and SOX2 after 30 days of exposure to ATZ (0.01, 1, and 10 µM) showed reduced expression and altered localization of both proteins across all hiPSC lines, with variation in pattern between lines (Figure 3a–c; WT, HD1, HD2). Quantification of ICC confirmed a significant decrease in OCT4 staining intensity (Figure 3d,e), particularly in HD1 and HD2 lines (*** *p* < 0.001). Similarly, qRT-PCR analysis showed downregulation of *OCT4* expression following ATZ exposure (Figure 3f,g), most prominently in HD cells treated with 10 µM ATZ, although changes were not statistically significant. In contrast, SOX2 staining intensity remained stable at 0.01 µM but was upregulated at 10 µM ATZ in all lines, while *SOX2* gene expression showed a mild reduction in HD cells.

### 3.4. The Impact of Atrazine on Human Spermatogenesis In Vitro

**Figure 4:**
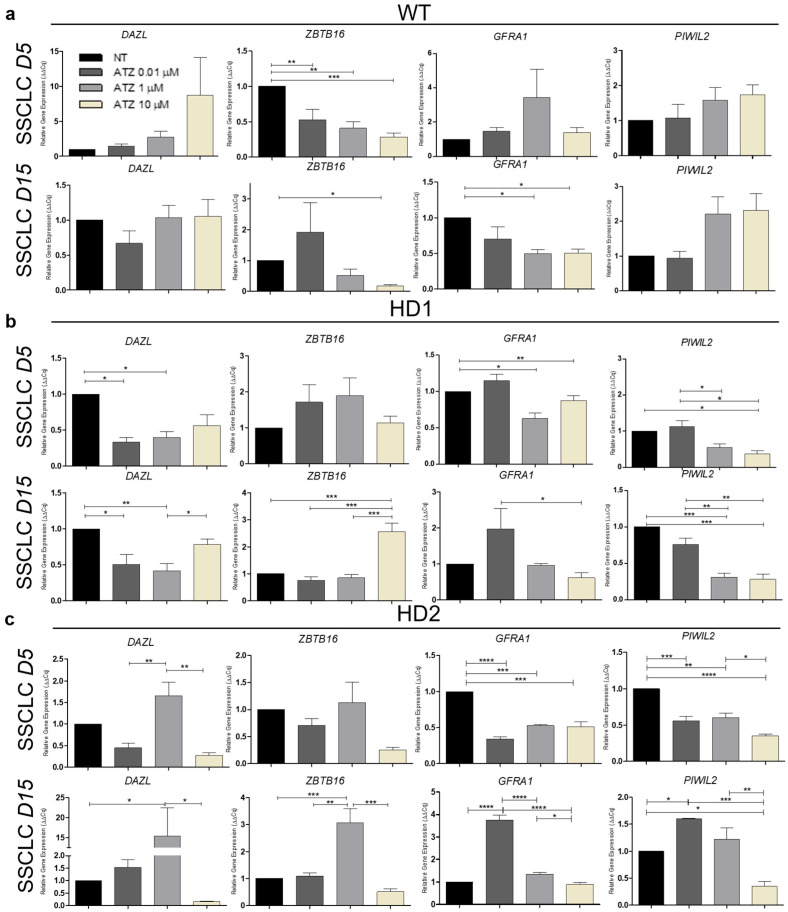

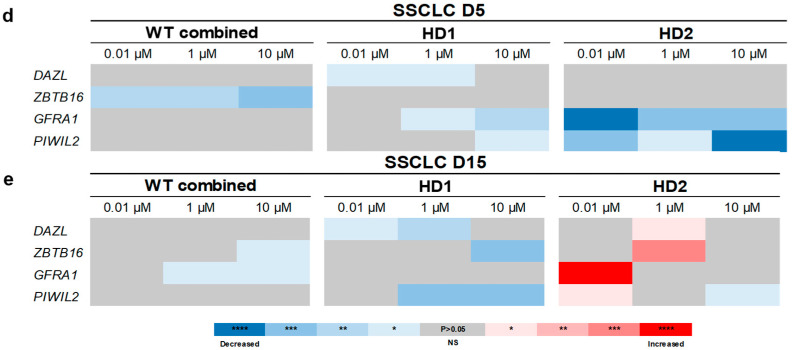
Effect of ATZ exposure during human spermatogenesis *in vitro*. (**a**–**c**) qRT-PCR analysis of relative gene expression of early SSC (*DAZL*, *ZBTB16*, and *GFRA1*) and late SSC (*PIWIL2*) markers at day 5 and day 15 of SSCLC differentiation in WT, HD1 and HD2, respectively. (**d**) Summary plot of SSC related gene expression at day 5 of SSCLC differentiation in WT, HD1 and HD2 compared to untreated control, respectively. (**e**) Summary plot of SSC related gene expression at day 15 of SSCLC differentiation in WT, HD1 and HD2, respectively. Gene expression is shown using a pseudo–color scale from red to blue. Red squares indicate significant upregulation, blue squares indicate significant downregulation, and gray squares indicate no significant difference (*p* > 0.05). Data are presented as mean ± SEM from three biological replicates (n = 3). Statistical significance was determined using one-way ANOVA followed by Tukey’s multiple comparison test: * *p* < 0.05, ** *p* < 0.01, *** *p* < 0.001, **** *p* < 0.0001.

To assess the effects of ATZ on human spermatogenesis, WT, HD1, and HD2 hiPSCs were exposed to ATZ (0, 0.01, 1, and 10 µM) for 30 days, followed by differentiation into SSCLCs for 15 days under continuous ATZ exposure (Figure 1a). Differentiation efficiency of differentiating cells was evaluated using ICC and qRT-PCR to monitor SSC marker expression. ICC showed no significant differences in germ cell-specific proteins (ZBTB16 and PIWIL2) at day 15 across all cell lines. In contrast, qRT-PCR revealed specific changes in SSC-related gene expression, exhibiting distinct sensitivity patterns among cell lines. In WT cells, ATZ caused a dose-dependent reduction in the early SSC marker *ZBTB16* at day 5 (*p* < 0.01–0.001), while *DAZL*, *GFRA1*, and *PIWIL2* remained largely unaffected. By day 15, *DAZL* and *PIWIL2* were comparable to controls, with slight induction of *PIWIL2* at 1–10 µM, whereas *ZBTB16* and *GFRA1* were significantly suppressed (*p* < 0.05), indicating sustained inhibition of early SSC markers without major effects on late-stage progression (Figure 4a,d,e). HD1 cells were highly sensitive to ATZ (Figure 4b,d,e). At 1 and 10 µM, *DAZL*, *GFRA1*, and *PIWIL2* were significantly reduced at day 5 (*p* < 0.05), suggesting impaired early SSCLC development. By day 15, *PIWIL2* remained low in all doses, with 10 µM strongly suppressing *GFRA1* (*p* < 0.05) and *PIWIL2* (*p* < 0.001) while *ZBTB16* remained elevated. These reflect impaired SSCLC maturation in HD1. Interestingly, HD2 cells exhibited a biphasic response (Figure 4c–e). At day 5, ATZ significantly reduced *GFRA1* and *PIWIL2*, indicating early-stage SSCLC disruption. At day 15, low-dose ATZ (0.01–1 µM) enhanced SSCLC progression, reflected by increased *DAZL*, *GFRA1*, and *PIWIL2* expression, whereas high-dose ATZ (10 µM) strongly suppressed all SSC markers, particularly *PIWIL2* (*p* < 0.05), suggesting potential apoptosis or impaired SSCLC differentiation.

Overall, these results demonstrate that ATZ exerts concentration-, stage-, and genotype-dependent effects on *in vitro* spermatogenesis. WT cells showed suppression limited to early SSCLC differentiation, HD1 cells displayed heightened sensitivity with impaired SSCLC maturation, and HD2 cells exhibited mixed, dose-dependent responses (Figure 4d,e). These findings underscore the complexity of ATZ’s impact on spermatogenesis and highlight the importance of considering genetic background, differentiation stage, and exposure conditions when evaluating reproductive toxicity *in vitro*.

### 3.5. Atrazine Impacts the Nrf2 Signaling Pathway During In Vitro Spermatogenesis

**Figure 5:**
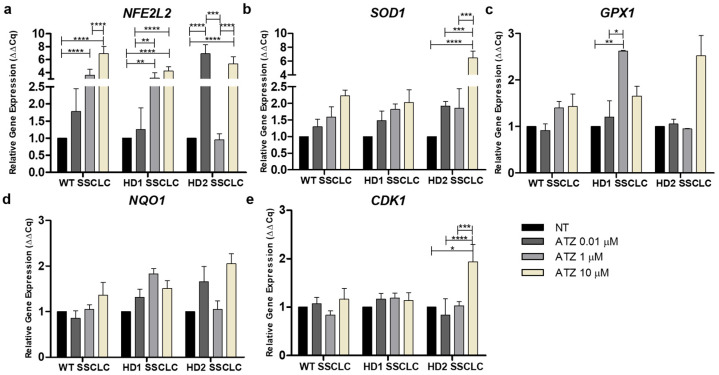
Effect of ATZ exposure on Nrf2 signaling pathway and cell cycle during IVS. (**a**) qRT-PCR analysis showing that ATZ treatment upregulated NFE2L2 expression in all SSCLC lines, indicating ATZ-initiated oxidative stress. (**b**–**d**) Relative gene expression of antioxidant defense–related genes (as *NFE2L2* target gene), including *SOD1*, *GPX1*, and *NQO1*, was also upregulated, particularly at 10 μM ATZ across all cell lines. (**e**) The expression of *CDK1*, a key regulator of the cell cycle and cell proliferation, remained stable in most groups, but showed a significant increase in HD2 SSCLCs treated with 10 µM ATZ. Data are presented as mean ± SEM from three biological replicates (n = 3). Statistical significance was determined using one-way ANOVA followed by Tukey’s multiple comparison test. Comparisons were made with the SSCLC no treatment of each cell line: * *p* < 0.05, ** *p* < 0.01, *** *p* < 0.001, **** *p* < 0.0001.

Building on our findings of altered SSC gene expression under ATZ exposure, and given that ATZ is known to induce DNA damage and oxidative stress via the Nrf2 signaling pathway, we next assessed whether ATZ modulates oxidative stress responses during SSCLC differentiation. The expression of *NFE2L2*, key antioxidant genes (*SOD1*, *GPX1*, *NQO1*), and the cell cycle regulator *CDK1* was examined in WT, HD1, and HD2 SSCLCs at day 15 (Figure 5a–e). In WT SSCLCs, ATZ induced a dose-dependent activation of Nrf2 signaling. *NFE2L2* expression increased significantly at 1 µM and 10 µM (*p* < 0.0001), with antioxidant genes showing upward trends but without statistical significance. At 1 µM, modest antioxidant induction coincided with *NFE2L2* upregulation, suggesting partial neutralization of ROS. At 10 µM, *NFE2L2* expression peaked (*p* < 0.0001) with stronger antioxidant induction, but a slight reduction in early SSC marker expression indicated interference with differentiation (Figure 4a). These findings suggest that WT cells mounted a sufficient antioxidant defense to buffer ATZ-induced stress, with only mild impairment of early SSC progression. HD1 SSCLCs carrying 44 CAG repeats, ATZ elicited a more stress-sensitive response. *NFE2L2* was strongly upregulated in a dose-dependent manner, with significant increases at 1 µM (*p* < 0.01) and 10 µM (*p* < 0.0001). *SOD1* expression followed this trend, peaking at 10 µM, while *GPX1* and *NQO1* were slightly elevated at 0.01 µM but sharply reduced at 10 µM. At 0.01 µM, incomplete antioxidant activation correlated with impaired SSC progression. At 1 µM, *NFE2L2* and *GPX1* upregulation suggested compensation, yet antioxidant capacity remained insufficient, consistent with impaired SSC maturation. At 10 µM, robust *NFE2L2* induction coincided with depletion of *GPX1* and *NQO1*, indicating excessive ROS consumption and impaired differentiation, as shown by marked suppression of *PIWIL2*. These findings highlight that in HD1 cells, strong *NFE2L2* activation alone was insufficient to restore redox balance under high-dose ATZ stress. In HD2 SSCLCs with over 180 CAG repeats, ATZ triggered a biphasic response. At 0.01 µM, *NFE2L2* was significantly upregulated (*p* < 0.0001), reaching its highest levels, alongside modest increases in *SOD1*, *GPX1*, and *NQO1*. At 1 µM, *NFE2L2* and antioxidant genes were slightly downregulated, yet SSC markers remained elevated, suggesting adequate antioxidant capacity to sustain differentiation. At 10 µM, both *NFE2L2* and antioxidant genes were strongly induced, with *SOD1* significantly upregulated (*p* < 0.0001). Notably, *CDK1* was also significantly elevated (*p* < 0.05 vs. control; *p* < 0.001 vs. lower doses), suggesting accelerated proliferation under oxidative stress (Figure 5e). This dysregulated profile likely predisposes cells to replication stress and DNA damage, consistent with impaired SSC maturation at high-dose exposure in HD2 cells.

Overall, across all genotypes, low-dose ATZ (0.01–1 µM) generally supported antioxidant defenses with only mild impacts on SSCLC differentiation. HD1 cells showed heightened vulnerability, where antioxidant depletion accompanied impaired SSC progression despite strong NFE2L2 activation. In contrast, high-dose ATZ (10 µM) consistently induced strong Nrf2 responses but disrupted SSC differentiation, with HD2 cells uniquely displaying CDK1 activation, indicative of proliferation-associated genomic stress. These findings demonstrate that ATZ-induced oxidative stress engages divergent adaptive responses depending on genotype, dose, and differentiation stage, ultimately shaping SSC developmental outcomes.

### 3.6. Atrazine Alters the Expression of Apoptotic Markers During In Vitro Spermatogenesis

**Figure 6:**
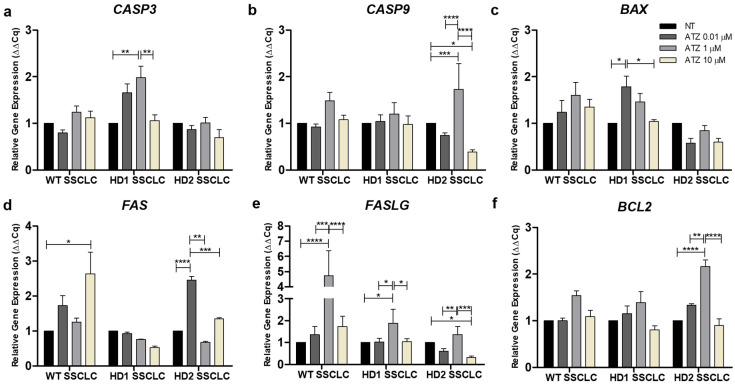
Effect of ATZ exposure on apoptosis in WT, HD1 and HD2 SSCLCs. (**a**–**e**) qRT-PCR analysis showing relative expression of pro-apoptotic genes (*CASP3*, *CASP9*, *BAX*, *FAS*, and *FASLG*, respectively). (**f**) Relative expression of the anti-apoptotic gene *BCL2*, showing a trend toward upregulation at 0.01 and 1 μM ATZ, followed by downregulation at 10 μM ATZ across all cell lines. Data are presented as mean ± SEM from three biological replicates (n = 3). Statistical significance was determined by one-way ANOVA followed by Tukey’s multiple comparison test. Comparisons were made with the SSCLC no treatment of each cell line: * *p* < 0.05, ** *p* < 0.01, *** *p* < 0.001, **** *p* < 0.0001.

To determine whether ATZ modulates apoptotic signaling in addition to activating Nrf2 responses, we examined the expression of pro-apoptotic genes (*CASP3*, *CASP9*, *BAX*, *FAS*, *FASLG*) and the anti-apoptotic gene *BCL2* in SSCLCs at day 15 of differentiation (Figure 6a–f). Across all cell lines, ATZ exposure altered apoptosis-related gene expression in a dose-dependent and genotype-specific manner.

In WT SSCLCs, *BAX* showed an upward trend at 0.01 µM, while *CASP3* and *CASP9* were slightly reduced. At 1 µM, both intrinsic and extrinsic apoptotic pathways appeared partially activated, reflected by increased *BAX*, *CASP3*, and *CASP9*, together with significant upregulation of *FASLG* (*p* < 0.01). At 10 µM, apoptotic markers declined toward basal levels, with only *FAS* significantly elevated (*p* < 0.05). *BCL2* showed a mild, non-significant increase across doses, suggesting preserved anti-apoptotic capacity. These results indicate that apoptosis was partially engaged at 1 µM but suppressed at higher doses, consistent with enhanced antioxidant buffering, leading to largely preserved SSCLC differentiation (Figure 4a). In HD1 SSCLCs, ATZ exposure induced dose-dependent changes in apoptosis-related genes (Figure 6a–f). At 0.01 µM, *BAX* was significantly induced (*p* < 0.05), with a slight increase in *CASP3*, while *FAS* and *FASLG* remained unchanged. *BCL2* did not change, suggesting limited anti-apoptotic defense. At 1 µM, *CASP3* (*p* < 0.01) and *FASLG* (*p* < 0.05) were significantly upregulated, accompanied by modest increases in *BAX* and *CASP9*, indicating partial activation of both intrinsic and extrinsic pathways. At 10 µM, pro-apoptotic markers declined to their lowest levels, despite strong *NFE2L2* induction and depletion of antioxidant genes (Figure 5a). This profile suggests suppression of apoptotic signaling under unresolved oxidative stress, correlating with impaired SSC progression and highlighting the heightened vulnerability of HD1 cells. In HD2 SSCLCs, ATZ produced a distinct response on apoptosis-related genes (Figure 6a–f). At 0.01 µM, *NFE2L2* and its antioxidant targets were strongly induced (Figure 5a), while *BCL2* increased slightly, and pro-apoptotic markers (*CASP3*, *CASP9*, *BAX*, *FASLG*) were reduced, except for *FAS*, which was significantly elevated. This pattern indicates effective antioxidant activation that suppressed apoptosis and supported SSC differentiation (Figure 4c). At 1 µM, *BCL2* was strongly upregulated (*p* < 0.0001), with only *CASP9* significantly increased (*p* < 0.001). This pattern suggests that ROS were effectively buffered by antioxidant defenses, thereby limiting full apoptotic activation while allowing differentiation to proceed. At 10 µM, *NFE2L2* and its antioxidant targets were strongly upregulated, whereas pro-apoptotic markers showed no significant changes (Figure 6a–e). *BCL2* was slightly reduced, while *CDK1* expression was notably elevated (Figure 5e). This response suggests that instead of apoptosis, oxidative stress promoted aberrant proliferation and possible replication stress, ultimately impairing SSC differentiation.

Overall, low to moderate ATZ doses (0.01 and 1 µM) partially activated intrinsic and extrinsic apoptotic pathways in both WT and HD1 cells, whereas higher doses (10 µM) attenuated apoptosis but impaired SSCLC differentiation. In contrast, HD2 cells showed pronounced oxidative stress with strong *NFE2L2*-driven antioxidant responses. SSC maturation capacity was largely preserved when antioxidant defenses were sufficient; however, excessive oxidative stress or proliferative signaling at high ATZ concentrations could compromise this process. These findings highlight the complex interplay between oxidative stress, Nrf2 activation, and apoptosis regulation during *in vitro* SSCLC differentiation, with outcomes determined by both ATZ dose and genetic background.

## 4. Discussion

Atrazine (ATZ) is a pervasive environmental contaminant, yet its impact on human spermatogenesis remains poorly understood, largely due to interspecies differences and the absence of a physiologically relevant human model. This knowledge gap is further pronounced in conditions such as Huntington’s disease (HD), where the limited availability of human samples has hindered detailed mechanistic studies. Previous reports have shown that alterations in sperm function occur in both animal models and human males with HD, suggesting that the mutant huntingtin protein may disrupt germline homeostasis [42,43,44]. Additionally, ATZ has been implicated in neurodevelopmental toxicity *in vitro* [45], inducing long-term epigenetic alterations and increasing the risk of neurodegenerative conditions such as Parkinson’s disease [46]. These overlapping toxicological and genetic susceptibilities underscore the importance of investigating ATZ effects using HD hiPSC-derived germ cell models. To explore these combined vulnerabilities, we used a human SSCLC differentiation model derived from WT and HD hiPSCs [35,37], enabling evaluation of 30-day ATZ exposure at environmentally relevant concentrations. The selected concentrations (0.01, 1, and 10 μM) span levels found in contaminated groundwater and doses previously shown to elicit cellular responses without inducing cytotoxicity [47,48,49]. This approach supports the interpretation of ATZ effects across realistic and experimentally validated exposure conditions.

Under control conditions, our SSCLC differentiation system successfully recapitulated stages of human spermatogenesis. All cell lines showed downregulation of pluripotency markers (*OCT4*) and upregulation of germ cell genes (*ZBTB16*, *DAZL*, *GFRA1*, *PIWIL2*) through day 15 of differentiation (Figure 1b,d), consistent with previous studies [35,38,50]. In addition, the differentiation protocol and hiPSC lines used here were previously validated by our group using RNA-seq, which showed clear enrichment of spermatogenesis-related pathways [37]. This independent evidence supports the reliability of our SSCLC generation method. Both HD-hiPSCs differentiated similarly to WT cells under untreatment conditions, suggesting that mutant *HTT* does not markedly impair early spermatogenic progression (Figure 2). *HTT* may primarily influence later stages via mRNA translation and chromatin regulation [51].

Because oxidative stress can impair stem cell function [41] and ATZ modulates gene expression in non-human primate pluripotent cells [52], we examined the effects of prolonged ATZ exposure on hiPSC pluripotency. Consistent with prior observations of *OCT4* downregulation at high ATZ concentrations (100 µM) [45], we observed significant downregulation in OCT4 staining intensity, particularly in HD1 and HD2 cells, indicating higher sensitivity of HD-hiPSCs likely due to compromised redox homeostasis. In contrast, SOX2 staining intensity was increased in hiPSCs treated with 10 µM ATZ, while *SOX2* gene expression remained largely unchanged (Figure 3e,g). This may represent a compensatory response to oxidative stress, as elevated ROS can induce *SOX2* expression to enhance antioxidant defenses and DNA repair, thereby maintaining stem cell identity [53,54,55,56]. Moreover, this observation aligns with the hypothesis that elevated SOX2 protein levels may activate a negative feedback loop that suppresses endogenous *SOX2* transcription [57]. In contrast, OCT4 appeared more sensitive to ATZ exposure, suggesting distinct regulatory mechanisms among the core pluripotency factors [58]. Environmentally relevant ATZ levels (0.01 µM) did not significantly alter OCT4 in WT cells, consistent with dose-dependent effects reported previously [6,8]. Overall, low ATZ exposure does not strongly disrupt hiPSC self-renewal, whereas higher or prolonged exposure, especially in HD lines may destabilize stemness and promote differentiation imbalance.

Oxidative stress has been implicated as a key mechanism underlying ATZ-mediated reproductive toxicity. There is extensive *in vivo* and *in vitro* evidence demonstrating that ATZ impairs testicular structure, reduces germ cell counts, and diminishes sperm quality [3,24], effects that have also been associated with prostate carcinogenesis [59]. Building on this evidence, we investigated whether ATZ modulates Nrf2 signaling and apoptotic pathways in human IVS. To mimic developmental exposure, hiPSCs were continuously treated with low doses of ATZ (0, 0.01, 1, and 10 µM) for 30 days prior to SSCLC differentiation. In our hiPSC-derived model, prolonged ATZ exposure impaired SSCLC differentiation through activation of oxidative stress and DDR pathways, particularly those involving Nrf2, cell-cycle control, and apoptosis signaling, as indicated by gene expression profiles, although protein-level validation remains a limitation.

**Figure 7:**
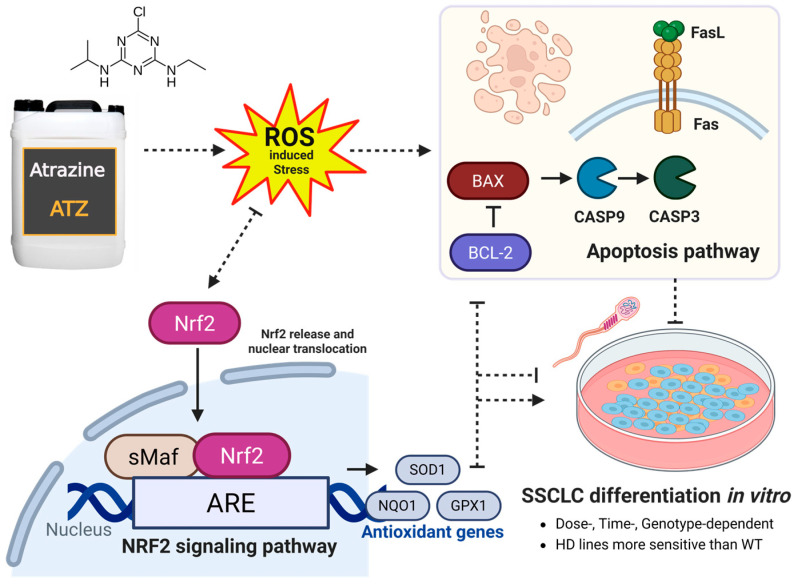
Summary of the effects of ATZ on *in vitro* spermatogenesis. A schematic summary illustrating the effects of ATZ exposure on SSCLC differentiation across all cell lines. ATZ influences the progression of *in vitro* SSCLC differentiation in a dose-, stage, and genotype-dependent manner, likely mediated through modulation of Nrf2 signaling, and apoptosis-related pathways, as suggested by gene expression profiles. The figure was created in BioRender. Chaiyakit M., Parnpai R., Cho I.K. (2025), https://app.biorender.com/illustrations/68e4d50e30d89ab383932282 (accessed on 16 October 2025).

These general effects differed among cell lines as summarized in Figure 7. In WT cells, environmentally relevant ATZ doses (0.01 µM) induced minimal effects, consistent with prior reports showing negligible cytotoxicity in normal human breast and prostate cells [15,60]. In contrast, 1 and 10 µM ATZ activated Nrf2 signaling and apoptotic pathways, altering SSC gene expression without fully inhibiting SSCLC differentiation. Moderate activation of apoptosis-related genes at 1 µM likely reflected residual oxidative stress insufficiently countered by Nrf2-driven antioxidant defenses. At higher doses (10 µM), stronger Nrf2 activation attenuated apoptotic signaling (Figure 5 and Figure 6), suggesting a compensatory feedback mechanism to restore redox balance. Similar dose-dependent effects have been reported *in vivo*, where ATZ reduced SOD activity while transiently maintaining CAT activity in testicular and epididymal tissues [61]. These findings indicate that WT cells maintain a relatively robust antioxidant capacity but remain vulnerable to oxidative dysregulation at higher ATZ concentrations.

Although SSCLC differentiation did not differ significantly between WT and HD cells under control conditions, HD cells had stronger and more variable responses under ATZ conditions. In HD1 cells, SSC-related gene expression declined dose-dependently, accompanied by increased Nrf2 and apoptotic gene activation (Figure 5 and Figure 6), suggesting oxidative stress exceeded cellular antioxidant capacity [5,62]. Both intrinsic (*BAX*, *CASP3*) and extrinsic (*FASLG*) apoptotic pathways were significantly triggered at 1 µM ATZ, while BCL2 remained unchanged, linking DDR activation to germ cell impairment (Figure 4b,e). At 10 µM, *BCL2* and pro-apoptotic genes (*CASP3*, *CASP9*, *BAX*, *FAS*, *FASL*) were slightly downregulated, whereas *NFE2L2* was markedly induced. Concurrent declines in *GPX1* and *NQO1* indicate depletion of antioxidant reserves, leading to suppressed apoptosis despite elevated oxidative stress. However, persistent ROS may impair spermatogenic progression, predisposing HD1 SSCLCs to redox imbalance, particularly in lines with expanded CAG repeats (manuscript in preparation). These observations are consistent with studies in human SH-SY5Y cells, which exhibit sensitivity to lower ATZ concentrations compared to HepG2 cells, yet tolerate higher levels in certain contexts [63], confirming cell-type- and dose-dependent sensitivity to ATZ. In HD2 cells, ATZ exposure elicited a biphasic response: low doses (0.01 and 1 µM) initially suppressed SSC genes expression at day 5 but partially recovered by day 15 (Figure 3c–e), whereas 10 µM ATZ markedly upregulated *CDK1*, a key cell cycle regulator. These results align with reports that short-term ATZ exposures could promote proliferation via ROS-mediated cell cycle acceleration [47,60,64]. The absence of apoptosis at 10 µM suggests abnormal proliferation, rather than cell death, predominates at higher doses, potentially contributing to DNA damage accumulation and impaired differentiation (Figure 3c, Figure 5 and Figure 6). Incomplete antioxidant defense and insufficient apoptotic clearance may allow defective germ cells to persist, consistent with fertility impairments observed in prior studies [65,66,67]. Collectively, these findings demonstrate that ATZ modulates SSCLC differentiation through oxidative stress and DDR signaling, with HD lines, particularly those carrying larger CAG repeats, showing heightened sensitivity. The combined effects of mutant huntingtin and ATZ-induced ROS likely exacerbate spermatogenic disruption by disturbing redox balance, DNA integrity, and apoptosis regulation.

In summary, our hiPSC-derived SSCLC differentiation model recapitulates key features of human spermatogenesis and provides a powerful *in vitro* platform for evaluating the impact of environmental toxicants. ATZ disrupted SSC development in a concentration- and genotype-dependent manner through activation of Nrf2 signaling and apoptosis pathways. These findings support the hypothesis that ATZ may exacerbate reproductive dysfunction and genomic instability, particularly in individuals predisposed to oxidative stress. Future investigations should further delineate the interplay between ATZ-induced ROS, DDR mechanisms, and trinucleotide repeat (TNR) instability to clarify how environmental exposures contribute to germline genome instability and infertility. Given its physiological relevance, this IVS model may also be applied in reproductive medicine to assess toxicant effects and develop strategies for mitigating male infertility.

## Data Availability

Data is contained within the article and Supplementary Materials.

## Supplementary Materials

The following supporting information can be downloaded at: https://www.mdpi.com/article/10.3390/biomedicines13122917/s1, Table S1: SYBR Green primer sequences.

## Abbreviations

The following abbreviations are used in this manuscript:

ATZ	Atrazine
DDR	DNA damage response
HD	Huntington’s disease
hiPSC	Human induced pluripotent stem cell
IVS	*In vitro* spermatogenesis
Nrf2	Nuclear factor erythroid 2–related factor 2 signaling pathway
NFE2L2	Nuclear factor erythroid 2–related factor 2 gene
ROS	Reactive oxygen species
SSC	Spermatogonial stem cell
SSCLC	Spermatogonial stem cell-like cell
TNR	Trinucleotide repeat
