# Supplementary material for: Atrazine Induces Reproductive Toxicity in an In Vitro Spermatogenesis (IVS) Model
Source: Biomedicines. 2025 Nov 28;13(12):2917. doi: 10.3390/biomedicines13122917 (PMC12731135; doi:10.3390/biomedicines13122917)
Supplement: Supplementary file 1 [file biomedicines-13-02917-s001.zip › biomedicines-3963907-Supplementary Materials.pdf]

biomedicines

MDPI

Supplementary Materials

Table S1. SYBR Green primer sequence.

Gene Symbol	Forward (5′-3′)	Reverse (5′-3′)
OCT4	GGAATGAGGGACAGGGGGAG	ACTCCCCTGCCCCCACCCT
SOX2	GCCGAGTGGAAACTTTTGTCG	GGCAGCGTGTACTTATCCTTCT
ZBTB16	GGACAAGGTTGAGGAAAGAGG	CAACACGGAGTAGATGCCCAG
DAZL	TTAAACTTCGAGGGCCGGAG	TTTGCAGTAGACTCGTGCCC
GFRA1	CCAAAGGGAACAACTGCCTG	CGGTTGCAGACATCGTTGGA
PIWIL2	AGGACTCGCGCACAGGTAAT	CTGAACGATGGTCGGAAAGGAT
FAS	AAGAAGACAAAGCCACCCCAA	AAGAAGACAAAGCCACCCCAA
FASLGL	GGCCTGTGTCTCCTTGTGAT	GGGTGGCCTATTTGCTTCTCC
CASP9	GGTGGGGAGCAGAAAGACC	AGCTGGTCGAAGGTCCTCAA
CASP3	TCGCTTTGTGCCATGCTGAAAC	TGTTGCCACCTTTCGGTTAACC
BAX	CAAACTGGTGCTCAAGGCCC	GCGTCCCAAAGTAGGAGAGG
BCL2	CGGGATGGGGTAAACTGG	AGGTGGTCATTCGTGG
NFE2L2	AGTCAGCGACGGAAAGAGTA	CGTAGCCGAAGAAACCTCAT
SOD1	AGCGAGTTATGGCGACGAAG	CAAGTCTCCAACATGCCTCTCT
NQO1	GAAAGGATGGGAGGTGGTGG	CTGCAGGGGGAACTGGAATA
GPX1	AGTCGGTGTATGCCTTCTCG	CTTGGCGTTCTCCTGATGCC
*GAPDH**	CCCACTGCCAACGTGTCA	AAGTCAGAGGAGACCACCT
